# Diagnostic Approaches to Cardiac Arrhythmias: Integrating Electrocardiography and Advanced Monitoring

**DOI:** 10.7759/cureus.111780

**Published:** 2026-06-29

**Authors:** Saidalavi Thengilan, Rakesh Venuturumilli, Naresh Sen, Ojas B Solanki, Kumar Sambhav, Naveed Mohsin

**Affiliations:** 1 Department of Cardiology, Government Medical College, Kozhikode, Kozhikode, IND; 2 Department of Critical Care and Extracorporeal Membrane Oxygenation, Fortis Gleneagles Hospital, Hyderabad, IND; 3 Department of Cardiology, Rama Medical College Hospital and Research Centre, Kanpur, IND; 4 Department of Forensic Medicine and Toxicology, Dr. N. D. Desai Faculty of Medical Science and Research, Dharmsinh Desai University, Nadiad, IND; 5 Department of Anatomy, All India Institute of Medical Sciences, Bilaspur, Bilaspur, IND; 6 Department of General Medicine, Sher-i-Kashmir Institute of Medical Sciences, Srinagar, IND

**Keywords:** ambulatory monitoring, arrhythmia diagnosis, electrocardiography, implantable loop recorder, wearable cardiac devices

## Abstract

Cardiac arrhythmias represent a broad spectrum of electrical disturbances and are associated with significant morbidity and mortality, necessitating accurate and timely diagnosis for effective management. Despite the central role of electrocardiography (ECG), limitations in capturing intermittent events and variability in interpretation create diagnostic gaps, particularly in patients with infrequent or asymptomatic presentations. This narrative review aims to synthesize current diagnostic approaches by integrating conventional ECG with advanced monitoring modalities, including ambulatory systems, implantable cardiac monitors, device-derived diagnostic data from pacemakers and implantable cardioverter-defibrillators, electrophysiological studies, imaging, and digital health technologies. Key findings indicate that while standard ECG remains indispensable for initial assessment, extended external monitoring and implantable cardiac monitors significantly improve detection rates, especially in sporadic arrhythmias. Additionally, diagnostic data from pacemakers and implantable cardioverter-defibrillators provide additional rhythm surveillance in patients with therapeutic cardiovascular implantable electronic devices. Multimodal integration, incorporating imaging and artificial intelligence-driven analytics, enhances diagnostic precision and supports personalized risk stratification. These developments have important clinical implications by enabling earlier detection, improving patient-specific management, and facilitating remote monitoring strategies. The findings highlight the need for structured, patient-centered diagnostic pathways that align modality selection with symptom frequency and clinical risk profiles. Continued technological innovation and data integration are expected to refine diagnostic accuracy and efficiency. Effective arrhythmia diagnosis relies on combining traditional and advanced tools within an integrated framework to optimize clinical outcomes.

## Introduction and background

Cardiac arrhythmias are a heterogeneous group of disorders caused by abnormal impulse formation, impulse transmission, or conduction within the heart. They may present as irregular rhythms, abnormal heart rates, or disorganized electrophysiological activity, ranging from benign premature beats to potentially fatal ventricular arrhythmias and sudden cardiac death. The clinical picture is broad, with cases being asymptomatic, palpitations, syncope, and hemodynamic instability, and therefore it is extremely challenging to diagnose and treat in the clinical setting [1]. Their clinical relevance has increased with population aging, the rising prevalence of cardiovascular risk factors, and improved survival among patients with structural heart disease [2]. Arrhythmias are often linked to some underlying conditions such as ischemic heart disease, cardiomyopathies, and heart failure, leading to the amelioration of morbidity, hospitalization, and health care expenses [3,4]. Moreover, the arrhythmic load in some patient groups, such as chemotherapy-induced cardiomyopathy patients or alcohol-induced cardiac dysfunction patients, is very high, hence the necessity of having accurate diagnostic techniques [5,6].

The mechanisms of arrhythmia are multifactorial and include abnormalities of impulse formation, such as enhanced automaticity or triggered activity, and abnormalities of impulse conduction, particularly re-entry circuits, which are common mechanisms of tachyarrhythmias [7]. Structural remodeling, myocardial fibrosis, ion channel dysfunction, and autonomic imbalance may create or worsen these electrophysiological substrates. Understanding these mechanisms is important because diagnostic findings must be interpreted in relation to the suspected arrhythmia mechanism, underlying cardiac substrate, and patient risk profile.

Electrocardiography (ECG) is the main diagnostic tool for arrhythmia because it is simple to obtain, is non-invasive, and has the capability to give a real-time evaluation of the electrical activity of the heart. The regular 12-lead ECG allows clinicians to identify rhythm and conduction abnormalities and indirect evidence of structural or ischemic heart disease. It is crucial in the distinction of supraventricular and ventricular arrhythmias, as well as in making immediate clinical decisions [1]. However, the standard 12-lead ECG has a brief recording window, which reduces its ability to capture intermittent, transient, or infrequent arrhythmic events occurring outside the acquisition period. This limitation has led to the use of ambulatory and advanced rhythm-monitoring strategies. The Holter monitoring, event recorders, and patch-based systems increase the time of monitoring of the rhythm, thus increasing the probability of capturing the intermittent arrhythmias. Most recently, long-term and continuous monitoring with high diagnostic sensitivity has been achieved with implantable loop recorders, as well as device-based diagnostics, including implantable pacemakers and implantable cardioverter-defibrillators (ICDs), when the cause of syncope is unestablished or in cases of cryptogenic strokes [8]. These modalities help correlate symptoms with electrical abnormalities and improve detection when arrhythmias are not present during routine ECG acquisition.

Digital health technologies have further expanded arrhythmia evaluation through wearable devices, photoplethysmography-based rhythm assessment, single-lead ECG recordings, and remote monitoring platforms. These devices have the capacity to screen large populations for arrhythmias, especially atrial fibrillation, and support remote patient monitoring. Nonetheless, the issues of data accuracy, false-positives, and clinical validation are still the subject of continuous research [9]. Besides non-invasive modalities, invasive electrophysiological studies (EPSs) offer comprehensive information about the mechanism of arrhythmia using intracardiac mapping and programmed stimulation. EPS has emerged as the gold standard for the diagnosis of complex arrhythmias and is extremely relevant for risk stratification and management. Integrating EPS findings with non-invasive ECG, ambulatory monitoring, imaging, and clinical assessment can support more precise diagnosis and individualized management [10].

With increased diagnostic options, it is essential that an integrative and comprehensive approach be used in evaluating patients with suspected arrhythmia. Selection of diagnostic tests should be based on clinical presentation, frequency of symptoms, symptom severity, baseline cardiovascular risk, and the likelihood of capturing an arrhythmic event during the selected monitoring period. In a practical patient-centered pathway, patients with ongoing symptoms or hemodynamic instability should first undergo immediate 12-lead ECG evaluation and urgent clinical assessment [1,10]. Patients with frequent daily or near-daily symptoms should be evaluated with short-term Holter monitoring, whereas those with weekly to monthly symptoms should be considered for extended external monitoring, including event recorders, external loop recorders, or patch-based monitors. Patients with infrequent symptoms, unexplained syncope, suspected silent arrhythmias, or cryptogenic stroke should be considered for longer-term monitoring with an implantable loop recorder. Invasive EPS should be reserved for patients with suspected complex supraventricular or ventricular arrhythmias, those with high-risk structural heart disease, those with unexplained syncope after non-invasive testing, or cases in which diagnostic clarification may directly guide catheter ablation or device therapy. This structured progression from standard ECG to extended monitoring, implantable monitoring, and invasive evaluation provides a clearer diagnostic framework for readers unfamiliar with arrhythmia workup while preserving patient-centered test selection. The purpose of this review is to make an extensive analysis of existing diagnostic methods of arrhythmias with emphasis on the integration of ECG with innovative monitoring tools. It will also outline the advantages and disadvantages of each test and explore trends in the future of diagnostics for arrhythmias.

Objectives of the review

This review aims to summarize conventional and advanced diagnostic techniques used in cardiac arrhythmia evaluation, with emphasis on ECG, ambulatory monitoring, implantable monitoring, electrophysiological evaluation, imaging, and digital health technologies. It discusses the advantages, limitations, and practical clinical applications of these modalities and explains how they can be integrated into a patient-centered diagnostic approach to improve arrhythmia detection and risk stratification.

Methodology

This comprehensive review was conducted to synthesize clinically relevant diagnostic approaches for cardiac arrhythmias and was structured to improve methodological transparency rather than to perform a formal systematic review or meta-analysis. Relevant literature was identified from PubMed/MEDLINE, Google Scholar, Scopus, Web of Science, and major cardiology and electrophysiology guideline sources. Search terms included cardiac arrhythmia diagnosis, ECG, 12-lead ECG, ambulatory ECG monitoring, Holter monitoring, patch monitor, external loop recorder, event recorder, implantable cardiac monitor, implantable loop recorder, EPS, wearable ECG, artificial intelligence ECG, remote monitoring, and cardiovascular implantable electronic devices. The review focused mainly on literature published from 2015 to 2026. Articles were considered when they addressed arrhythmia mechanisms, diagnostic modality selection, monitoring yield, rhythm surveillance, risk stratification, or integration of conventional and advanced diagnostic tools in adult cardiac arrhythmia care. Sources were not retained when they were unrelated to cardiac arrhythmia diagnosis, focused only on treatment without diagnostic relevance, lacked accessible full text or sufficient methodological detail, involved non-cardiac rhythm disorders, or duplicated information already covered by stronger contemporary evidence. Because this was a comprehensive narrative review, formal PRISMA study screening, quantitative synthesis, and individual-study risk-of-bias scoring were not performed; however, source selection emphasized relevance, recency, guideline authority, and clinical applicability. No quantitative synthesis, meta-analysis, meta-regression, pooled effect estimate, P-value calculation, or confidence interval reporting was performed because the review did not combine numerical outcomes across studies. Therefore, statistical peer review was not required. The review was organized into a clinically logical pathway from initial ECG assessment to short- and long-term monitoring, implantable cardiac monitors, device-derived rhythm data, electrophysiological evaluation, imaging, and digital diagnostics.

## Review

Fundamental principles of arrhythmia diagnosis

The application of diagnostic instruments is the basis of the diagnosis of cardiac arrhythmias. The impairment in the production or transmission of impulses is a cause of arrhythmias, and a model is needed to relate the underlying processes to the clinical and electrocardiographic outcomes [1,7]. One of the most important principles is to separate the abnormalities of automaticity, triggered activity, and re-entry circuits because all of the aforementioned mechanisms are manifested by the normal electrical activity and clinical significance [7]. In the diagnosis of arrhythmia, clinical context still plays a pivotal role. Symptom characterization, such as palpitations, dizziness, syncope, or no symptoms, affects the modalities of the diagnosis and the pre-test probability [9]. Of interest is the time relations between the symptoms and arrhythmic events that, in intermittent arrhythmias, are frequently difficult to detect in the absence of long-term monitoring. Additionally, there are also structural heart disease or systemic conditions that are also important contributors to the risk of arrhythmia and diagnostic priorities [3,4]. The electrocardiographic examination forms the basis of the diagnosis and enables the identification and description of the abnormalities of the rhythm on the basis of the morphology, rate, and patterns of the conduction of the waveforms. Although useful, the traditional interpretation of the ECG is not necessarily sensitive to detect transient events, and the introduction of monitoring measures is necessary [1,10]. More advances in computational analysis and electrophysiology have enhanced the accuracy of the diagnosis by enhancing the interpretation of the signal and correlation of electrical activity and pathophysiology [11,12]. It should be understood that a diagnostic strategy is, therefore, a combination of the physical exam, ECG interpretation, and the use of monitoring devices. This multi-dimensional concept is implemented because of the need to accurately diagnose the specific kind of heart problem, the causes, and the risks associated with it.

Initial assessment with standard electrocardiography

The main instrument in the diagnosis of cardiac arrhythmias is the standard 12-lead ECG, which is a non-invasive, fast measurement of cardiac electrical activity. It enables the identification of rhythm anomalies, conduction disorders, and indirect evidence of structural or ischemic heart disease through the systematic analysis of the waveforms, intervals, and axis aberrations [1]. P-wave morphology, PR interval, QRS duration, and QT interval are key parameters that are important in the differentiation between supraventricular and ventricular arrhythmias and in the diagnosis of conduction system disease. ECG has been most reliably diagnosed in the case of typical arrhythmias, such as atrial fibrillation, and the typical features of the absence of discrete P waves and irregular RR intervals. When properly interpreted, systematic analyses have shown high sensitivity and specificity of the 12-lead ECG in the detection of atrial fibrillation [10]. In addition, ECG can detect acute ischemia, electrolyte imbalances, and hereditary channelopathies, which could predispose to arrhythmogenesis [7,9].

Despite being central, there are limitations to conventional ECG. It has a short recording time, which decreases the chances of recording transient or paroxysmal arrhythmias and results in the possible underdiagnosis of symptomatic patients with rare episodes [1,3]. Besides, the variability of interpretation and the experience of clinicians can affect diagnostic consistency. ECG interpretation systems that are computer-assisted and can improve the efficiency of the diagnostic process have been created, but they can also give inaccurate results and thus must be validated by an expert [13]. The computational methods and digital signal processing have improved the analysis of ECG, making it possible to detect the smallest abnormalities and combine with the current diagnostic processes [14,15]. Nevertheless, the initial and inevitable stage of the assessment of arrhythmia is a routine ECG, and this forms the foundation of further utilization of other advanced monitoring modalities.

Advanced electrocardiographic techniques

Contemporary ECG has amplified the diagnostic capability of the simple ECG to clarify the signal more to enable the detection of small electrical abnormalities pertaining to arrhythmogenesis. These techniques are adjunctive rather than routine first-line diagnostic tools and are most relevant in selected patients with structural heart disease, suspected ventricular arrhythmia substrates, or inconclusive findings on standard ECG and ambulatory monitoring [1,7]. Signal-averaged electrocardiography (SAECG) is a specialized non-invasive ECG technique that enhances the detection of low-amplitude late potentials, which may reflect delayed ventricular activation, slow conduction zones, and potential re-entry substrates. Its historical and investigational use has been most commonly described in post-myocardial-infarction patients, patients with ischemic heart disease undergoing ventricular tachycardia assessment, and selected patients being evaluated for ventricular arrhythmia risk [16]. SAECG reduces background noise by averaging multiple cardiac cycles, thereby allowing the detection of microvolt-level abnormalities that are not visible on conventional ECG recordings. Its contemporary clinical role is limited because its incremental prognostic value beyond left ventricular function, cardiac magnetic resonance-based scar assessment, genetic risk markers, and other modern risk-stratification tools remains uncertain. Reported diagnostic performance for SAECG has varied across populations, acquisition methods, and outcome definitions; therefore, its sensitivity and specificity are not sufficiently consistent to support broad stand-alone clinical decision-making, and results should be interpreted only in conjunction with clinical findings, ventricular function, imaging, and other risk markers [16,17].

Spatial and temporal analysis of cardiac electrical activity is further refined by high-resolution ECG and vectorcardiography. Such techniques provide a more descriptive definition of the patterns of depolarization and repolarization, allowing to describe the arrhythmic substrates and conduction heterogeneity more effectively [17,18]. Similar to SAECG, these modalities are generally used in specialized clinical or research settings rather than as universal screening tests. Their application is most appropriate when conventional ECG and routine ambulatory monitoring are insufficient to characterize the suspected arrhythmic substrate. These techniques are progressively finding application in research and in special clinical settings, particularly in the evaluation of complicated arrhythmias.

The latest tendencies in the computational ECG analysis involve the implementation of more advanced algorithms capable of automatically classifying the rhythm and morphologically assessing it. The machine learning and signal processing techniques have significantly enhanced the precision of the diagnostics and have allowed for the detection of arrhythmias at an early stage and to predict the negative outcomes [14,15]. The innovations are helpful in clinical decision-making as they reduce the interpretation variability and increase efficiency. Regardless of these developments, the advanced ECG techniques are still used as a supplement to conventional ECG in clinical practice. Their use should be guided by the clinical question, suspected arrhythmia mechanism, patient risk profile, availability of complementary diagnostic tools, and whether the result is likely to produce an actionable change in management. Table 1 summarizes the electrocardiographic modalities in the diagnosis of arrhythmia.

**Table 1:** Electrocardiographic modalities in arrhythmia diagnosis AI, artificial intelligence; ECG, electrocardiography, SAECG, signal-averaged electrocardiography

Modality	Duration of Monitoring	Key Features	Clinical Applications
Standard 12-lead ECG	Seconds	Immediate rhythm assessment is widely available	Initial diagnosis: acute arrhythmias
SAECG	Minutes	Detection of late potentials, enhanced sensitivity	Ventricular arrhythmia risk stratification
High-resolution ECG	Minutes	Improved spatial and temporal resolution	Complex arrhythmia evaluation
Computational ECG analysis	Variable	AI-based interpretation, automated detection	Screening and diagnostic support

Short-term ambulatory monitoring

Ambulatory recording of electrocardiograms is very important in the process of detection and assessment of cardiac arrhythmias not recorded in the routine 12-lead ECG. Among these modalities, the most widely used has been Holter monitoring, which captures cardiac rhythm in an uninterrupted period of 24-72 hours in traditional systems, although extended Holter devices may record for longer periods depending on the device type, institutional protocol, and clinical indication. Using the approach, the symptoms can be correlated with temporary arrhythmia episodes, and the frequency, duration, and circadian variability of arrhythmia can be measured [1].

Holter monitoring is an appropriate first ambulatory choice when symptoms occur daily or very frequently because the probability of capturing the rhythm during a short continuous recording period is highest in this setting. It should not be regarded as universally the most effective modality since diagnostic yield depends on symptom frequency, symptom duration, baseline cardiovascular risk, and the diagnostic endpoint being assessed [19,20]. The traditional 24- to 72-hour Holter monitoring is most useful for frequent palpitations, dizziness, suspected frequent ectopy, or the assessment of heart-rate variability and arrhythmia burden. Extended Holter systems may provide longer continuous recordings and can improve detection when events are less frequent than daily but still occur within the monitoring window. Patch recorders generally provide longer, more comfortable continuous monitoring, often over 14-30 days and may be preferable when intermediate-duration monitoring is needed. Patient-activated event recorders are useful when patients can recognize symptoms and trigger recordings, whereas external loop recorders store pre- and post-event rhythm data and are more suitable for intermittent or unpredictable symptoms. Mobile cardiac outpatient telemetry allows near-real-time rhythm transmission and may be useful in higher-risk patients or when rapid clinician notification is clinically important [19-22]. It may also apply to the diagnosis of silent arrhythmia, such as asymptomatic atrial fibrillation, which may have severe clinical outcomes, including the risk of increased stroke [18]. Additionally, it provides quantitative data regarding the variability in heart rate and ectopic load, which can be applied in the risk stratification and treatment decisions [7,9].

The infrequent or sporadic symptoms in patients give a low diagnostic yield with short-term monitoring despite its utility. It is also demonstrated that the probability of clinically significant arrhythmia recording is much smaller in the case of events occurring outside the monitoring period [19,20]. Comparative evidence indicates that longer monitoring strategies generally increase diagnostic yield compared with short-duration Holter monitoring when symptoms are intermittent or unexplained, although this benefit must be balanced against cost, data burden, patient adherence, and clinical urgency [19-21]. This shortcoming usually requires the extended or implantable monitoring strategies to enhance better detection. Advances in technology over the last few years, including digital storage, automated analysis algorithms, improved electrode design, wireless data transfer, and remote review platforms, have expanded the range of applications of the Holter and enhanced the comfort of the patient. However, the issue of patient compliance, artefact effect of the electrodes, and a vast amount of data to be processed by the professionals remain [21]. This means that, depending on the clinical history and the frequency, duration, and risk profile of symptoms, the short-term ambulatory measurement is a useful stepping stone between the classical ECG and the long-term ambulatory monitoring modalities and can constitute valuable diagnostic information.

Long-term external monitoring systems

Long-term external monitoring has been extremely helpful in the diagnostic evaluation of intermittent and infrequent cardiac arrhythmias that are not identified by short-term monitoring. Ability to monitor the rhythm is enhanced to weeks to months with external event recorders, loop recorders, and patch-based, long-term monitoring systems. Their key advantage is that they can record transient arrhythmia events and establish a time correlation between arrhythmia and symptoms that patients report [1]. The event recorders can either be patient-initiated or can be activated to record at the time of symptoms, or when indicators of pre-defined arrhythmic patterns are observed. ECG data are stored and overwritten on external loop and continuous recorders and preserve segments before and after activation, thereby increasing diagnostic yields in sporadic arrhythmias [21]. The gadgets prove particularly useful in a patient with unexplained palpitations or syncope at a lower frequency than may be detected during a Holter monitoring [19,20].

Patch-based monitoring systems are a more recent technology that offers non-wireless and continuous ECG recording, with higher patient compliance and comfort. They can record long periods of time, up to a few weeks, and can be used to send data outside of the clinic, which makes the devices more accessible and easier to operate in clinical practice [22]. They possess a low motion artifact and enhanced signal quality as compared to the conventional electrode designs. Connection to internet health systems has further increased the functions of long-term monitoring, which makes it possible to exchange real-time data and manage the clinic remotely. The implementation of patient-centered health record systems and relevant models of monitoring allows more and improved patient engagement and compliance with the long-term monitoring processes [23].

**Figure 1:**
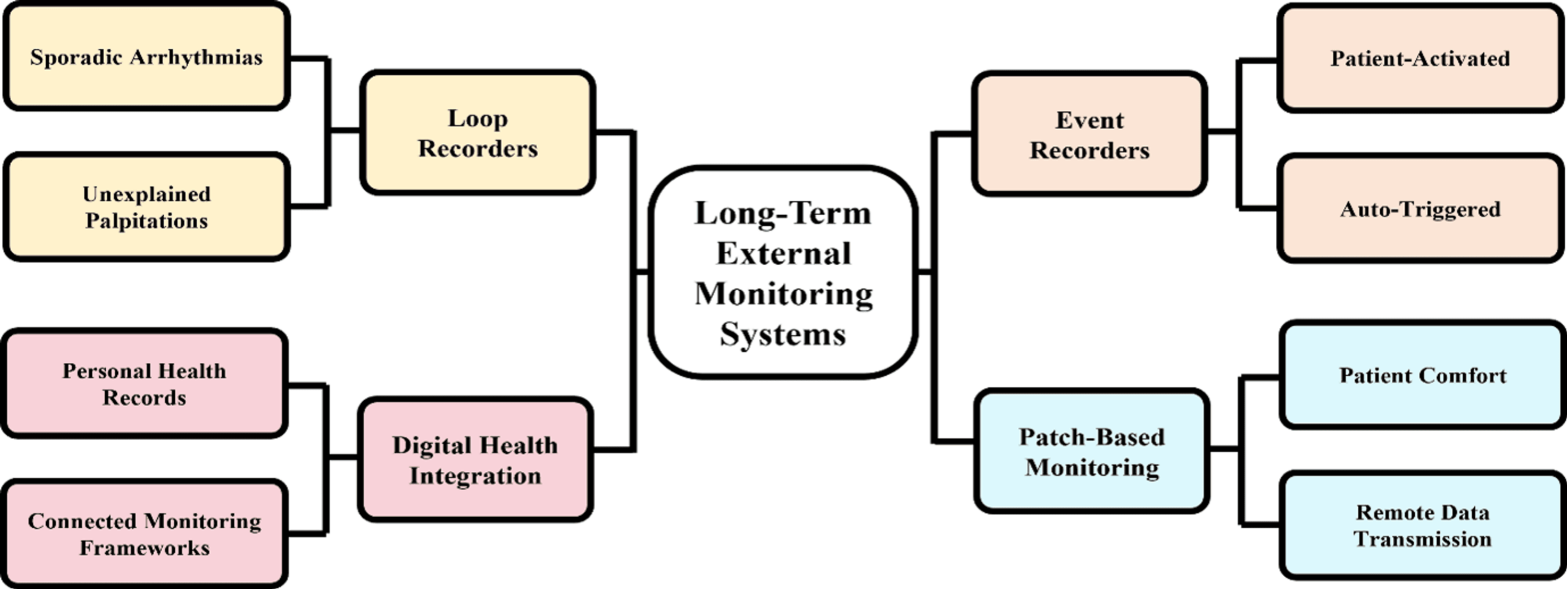
Long-term external cardiac monitoring systems Figure created by authors using Microsoft PowerPoint (Microsoft Corp., Redmond, WA)

Despite these benefits, there are still certain challenges, including data overload, inconsistent patient compliance, and the need to effectively analyze data. Nonetheless, long-term external monitoring systems might be significant in closing the gaps between the short-term monitoring and implantable devices in a full diagnostic procedure of cardiac arrhythmias. Figure 1 shows the external monitoring systems that will be used long-term to diagnose arrhythmia, which include event recorders, loop recorders, patch-based monitoring, and digital health integration.

Implantable cardiac monitors and device-derived diagnostic data

Implantable loop recorders, also called implantable cardiac monitors, are primarily diagnostic devices for long-term rhythm surveillance and should be distinguished from ICDs, which are therapeutic cardiovascular implantable electronic devices that can detect and treat selected ventricular tachyarrhythmias. Implantable cardiac monitors provide continuous subcutaneous ECG recording for months to years and are especially useful in patients with unexplained syncope, cryptogenic stroke, suspected silent atrial fibrillation, or infrequent palpitations not captured by external monitoring [24,25]. Their main advantage is prolonged automatic rhythm detection without reliance on patient activation or external electrodes, although use requires careful patient selection because of cost, implantation requirements, and procedural or device-related risks [24-26].

Pacemakers and ICDs may provide diagnostic information, including stored electrograms, arrhythmia burden, atrial or ventricular arrhythmia detection, and device alerts, but they should not be grouped with implantable cardiac monitors because their primary role is therapeutic pacing or defibrillation [27]. Remote monitoring can transmit rhythm data, device alerts, and stored arrhythmic episodes from implantable cardiac monitors, pacemakers, and ICDs, supporting earlier clinical review and reducing reliance on frequent in-person device interrogation [28]. Overall, implantable cardiac monitors are appropriate when non-invasive monitoring is insufficient for diagnosis, whereas pacemaker- and ICD-derived diagnostic data are adjunctive rhythm surveillance tools in patients who already require or possess therapeutic cardiovascular implantable electronic devices. Comparisons of ambulatory monitoring systems, implantable cardiac monitors, and device-derived diagnostic data sources are given in Table 2.

**Table 2:** Ambulatory monitoring, implantable cardiac monitors, and device-derived diagnostic data sources AF, atrial fibrillation; CIED, cardiovascular implantable electronic device; ECG, electrocardiography; ICD, implantable cardioverter-defibrillator; ICM, implantable cardiac monitor; ILR, implantable loop recorder

Monitoring/Data Source	Typical Duration	Primary Function	Key Diagnostic Use	Appropriate Clinical Context
Traditional Holter monitor	24-72 hours	External continuous ECG monitoring	Symptom-rhythm correlation, ectopy burden, heart-rate variability	Daily or very frequent symptoms
Extended Holter or patch-based monitor	Several days to 14-30 days, depending on device and protocol	Extended external ECG monitoring	Improved detection of intermittent arrhythmias compared with short-duration Holter monitoring	Intermediate-frequency symptoms
Patient-activated event recorder	Weeks	Patient-triggered external rhythm recording	Documentation of rhythm during recognized symptoms	Intermittent symptoms with patient awareness
External loop recorder/mobile cardiac outpatient telemetry	Weeks	External loop recording or near-real-time telemetry	Pre-/post-event rhythm capture and/or rapid data transmission	Unpredictable symptoms or higher-risk patients needing timely review
Implantable cardiac monitor/implantable loop recorder	Months to years	Primary diagnostic long-term subcutaneous ECG monitoring	Detection of infrequent, unexplained, or asymptomatic arrhythmias	Unexplained syncope, cryptogenic stroke, infrequent palpitations, suspected silent atrial fibrillation
Pacemaker/ICD-derived diagnostic data	Continuous while device is implanted	Therapeutic cardiovascular implantable electronic device with diagnostic monitoring functions	Stored electrograms, arrhythmia burden, atrial and ventricular arrhythmia detection, device alerts	Patients with existing indications for pacing or ICD therapy; not a primary diagnostic monitor equivalent to an implantable cardiac monitor

Invasive electrophysiological evaluation

EPS is a thorough diagnostic tool used in diagnosing complex heart rhythm disturbances, especially when non-invasive techniques fail to provide a conclusive diagnosis. IEC is a procedure using intracardiac catheters to evaluate the electrical activity, conduction systems, and arrhythmia inducibility in a controlled setting. It is used to gain insight into the mechanisms of arrhythmogenesis and can detect abnormal conduction pathways and arrhythmia substrate [29]. EPS has been particularly beneficial in the diagnosis of supraventricular tachycardia, ventricular tachyarrhythmia, and syncope of unexplained etiology. It allows distinguishing between re-entrant and focal mechanisms and evaluating the properties of the conduction system, such as refractory periods and conduction velocities [1,7]. Moreover, EPS is important in the risk stratification of patients who have structural heart disease or those who are at risk of sudden cardiac death [3,4].

**Figure 2:**
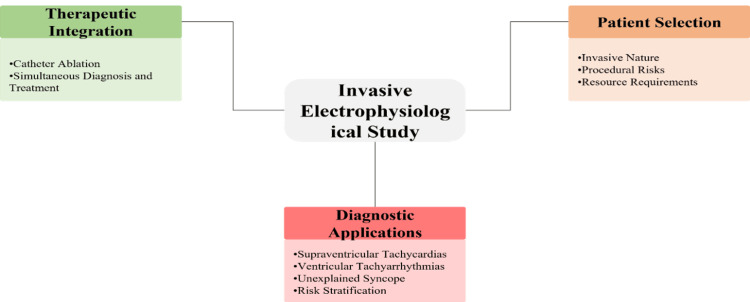
Role of invasive electrophysiological study in the diagnosis and management of cardiac arrhythmia Figure created by authors using Microsoft PowerPoint (Microsoft Corp., Redmond, WA)

Improvements in cardiac mapping technologies have greatly improved the diagnostic accuracy of EPS. Three-dimensional electroanatomical mapping systems and high-density mapping systems allow the visualization of the arrhythmic circuits in a more detailed manner, as well as identification of arrhythmogenic substrates at a more detailed level [30,31]. Alternative non-invasive mapping methods have been developed that complement the information and ease the complexity of the process in some cases [32]. Therapeutic interventions such as catheter ablation are usually used together with EPS, which allows arrhythmias to be diagnosed and treated at the same time. However, its invasive nature, risks associated with the procedure, and consumption of resources predetermine the necessity to be selective in terms of the type of patients that should be subjected to it. It should be noted that, generally speaking, invasive electrophysiology tests represent one of the fundamental aspects of the diagnostic process, especially in cases where there are complicated or resistant arrhythmias, as they do not offer any unique information on cardiac electrophysiology but instead help determine the application of specific therapy (Figure 2).

Imaging and multimodal risk stratification

The combination of cardiac imaging techniques and digital health technology has been extremely helpful in evaluating cardiac arrhythmias by providing structural, functional, and electrophysiological information. Although the electrocardiographic technique is central to the evaluation of rhythm abnormalities, the imaging technique, such as echocardiography, cardiac MRI, and CT scans, is used to identify any underlying substrate that can lead to arrhythmogenesis [1,7]. Myocardial fibrosis, chamber dilation, and ischemic changes, which are important to identify the risk and diagnose arrhythmia, are detectable using these imaging techniques [3,4]. The diagnostic accuracy has also been improved, thanks to some recent advancements in the sphere of multimodal imaging integration. The use of echocardiography, CT, and MRI enables visualization of a broad spectrum of cardiac structures and their tissue characterization, which makes it possible for the correct localization of substrates of arrhythmogenesis and further therapy [33]. In addition, images obtained by different imaging techniques and electrophysiological mapping can be used for the assessment of the treatment success as well as for pre-procedure planning in complex arrhythmia cases [30,31].

The digital health technologies that have pushed the boundaries of arrhythmia detection are wearable devices and remote monitoring platforms that allow for arrhythmia measurement away from the healthcare environment. These devices can continuously monitor cardiac rhythm in real time, facilitate early detection of asymptomatic arrhythmias, and enhance patient engagement in disease management [9,22]. Moreover, the multimodal data might be more precisely diagnosed, and the risk prediction might be more personalized with the assistance of the developed computational frameworks and machine learning systems [34,35]. With all these developments, data integration complexity, lack of similarity in data quality, and standardized clinical processes remain. The innovation of multimodal integration, however, cannot be underestimated in the diagnosis of arrhythmia since it will be a more holistic and accurate method of assessing the patient.

Wearable technologies and emerging digital diagnostics

These wearable devices have revolutionized the entire paradigm of detecting and diagnosing cardiac arrhythmia since it is capable of measuring the cardiac rhythm in real-time and continuously, even without being in the clinic. Devices such as smartwatches, fitness trackers, and portable ECGs are used for detecting irregular heart rhythms using the PPG and single-lead ECG because it provides higher accuracy [9,10]. Due to high availability, these devices have been used on a large number of patients, leading to the early diagnosis of asymptomatic cardiac arrhythmia that could not be detected with conventional diagnostic approaches. This is feasible through integrating the wearables with mobile applications in health care to transfer the data without interruption, remote monitoring, and acting quickly. This strategy facilitates more interaction with the patient and assessing the impact of arrhythmias over a long period of time, which is critical in risk stratification and maximizing treatment benefits [22,23]. In addition, artificial intelligence and computer algorithms improve signal processing and automatic rhythm detection by automatically analyzing the data and improving the accuracy of the diagnoses [14,15].

These devices can also be considered as an alternative to traditional and implantable monitoring tools for arrhythmia surveillance using a noninvasive approach to monitor their condition for a long period of time. This technique may be useful in individuals experiencing unpredictable symptoms or those who require surveillance in the aftermath of treatment procedures [19,25]. Also, the integration with other sources of data, including images and electrophysiology, improves diagnostic precision and encourages individualized treatment techniques [36,37]. These strengths notwithstanding, some challenges that can be identified include false-positives in identification, differences in the accuracy of the device, and the inability to standardize the data. Nevertheless, wearable and digital diagnostic devices can be viewed as a new field that holds immense promise in revolutionizing the field of arrhythmia management.

Limitations and future directions

The present-day technology utilized for cardiac arrhythmia diagnostics presents many limitations, regardless of the rapid technological development. The traditional ECG presents low sensitivity as a result of lower sensitivity during short periods of recording time for irregular arrhythmias. Long-term monitoring and ambulatory devices can be impacted by patient compliance issues, interference from the signal, and large amounts of data that have to be interpreted. Implantable technology proves highly effective; however, it entails higher costs and additional risks and is less commonly used. Moreover, there are challenges with clinical implementation in terms of wearables and varying diagnosis accuracy, as well as the risk of false-positives.

The future of arrhythmia diagnostics will be influenced by the further development of precision, effectiveness, and personalization of diagnostics. The AI will enhance the ECG interpretations, as will the machine learning algorithms. Predictive analytics will also be integrated into the diagnosis process and will enable earlier detection of arrhythmia. Stratification and personalized management will be facilitated through multimodal diagnostic approaches and information gained from ECG, imaging, and genomics. It will enable more convenient and continuous monitoring, and it is going to need a general clinical framework and data model to enhance monitoring efficiency, using new wearable and remote technologies. model to enhance monitoring efficiency, using new wearable and remote technologies.

## Conclusions

Diagnostic evaluation of cardiac arrhythmia is a complex procedure that includes clinical evaluation and a series of electrocardiographic and advanced testing systems. The 12-lead ECG remains the most important initial diagnostic test and provides essential information regarding rhythm and conduction abnormalities. However, because the standard 12-lead ECG has a brief sampling window, it may not capture intermittent, transient, or infrequent arrhythmic events occurring outside the recording period; therefore, ambulatory monitoring, extended external monitoring, and implantable cardiac monitors may be needed to complement diagnostic evaluation in patients with rare, intermittent, or silent arrhythmias. Diagnostic data from pacemakers and ICDs may also support rhythm surveillance, but these therapeutic cardiovascular implantable electronic devices should be distinguished from implantable cardiac monitors. With these technologies, including electrophysiological techniques, imaging, and digital healthcare solutions, the mechanisms of arrhythmia and the underlying structural bases can be better understood. The introduction of wearables and artificial intelligence-assisted predictive analytics has extended arrhythmia diagnosis beyond the clinic, allowing long-term monitoring and earlier intervention. The use of diagnostic tools matched to each case, depending on symptom frequency, symptom characteristics, and clinical risk, can improve diagnostic yield and patient-specific management. Ongoing development of multimodal data integration may help make cardiac arrhythmia diagnostics more efficient and personalized.
